# Retention and characteristics associated with remote questionnaire completion in a general population cohort study: the project baseline health study

**DOI:** 10.3389/fdgth.2025.1520132

**Published:** 2025-06-24

**Authors:** Megan K. Carroll, Safa Faheem, Jean Bouteiller, Adrian Hernandez, Kenneth W. Mahaffey, Jessica L. Mega, Neha Pagidipati, Terry Schaack, Svati H. Shah, Sumana Shashidhar, Susan Swope, Donna Williams, R. Scooter Plowman, Edgar P. Simard, Sarah A. Short, Shannon S. Sullivan

**Affiliations:** ^1^ Verily Life Sciences, South San Francisco, CA, United States; ^2^ Duke University, Durham, NC, United States; ^3^ Stanford Department of Medicine, Stanford University School of Medicine, Stanford, CA, United States; ^4^ California Health & Longevity Institute, Westlake Village, CA, United States; ^5^ Department of Pediatrics, Division of Pulmonary, Asthma and Sleep Medicine, Stanford University School of Medicine, Stanford, CA, United States

**Keywords:** research participant engagement, participant retention, remote study, cohort study, patient-reported outcomes, social determinants of health, project baseline health study, PBHS

## Abstract

**Objective:**

To evaluate remote participant engagement in a clinical study over time, based on data from the Project Baseline Health Study (PBHS), a hybrid in-person and virtual study.

**Methods:**

The PBHS enrolled 2,502 adult US residents from March 3, 2017 to April 26, 2019, with a ≤5-year follow-up. We summarized 4-year retention and rates of longitudinal patient-reported outcome survey completion. We investigated participant characteristics for their associations with quarterly remote survey completion using regression models.

**Results:**

Of the total participants (*N* = 2,502), 94% remained enrolled after 4 years and 60% completed all annual visits; 2,490 participants stayed enrolled for at least one quarter. The median (IQR) number of remote electronic survey sets completed was 8 (3–12), of a possible 16. Age [odds ratio (OR), >70 vs. ≤30 years: 2.56; 95% CI: 2.24–2.94] and education (OR, advanced degree vs. ≤high school: 1.36; 95% CI: 1.22–1.52) were positively associated with remote survey completion. Participants with lower odds of completion were Black (OR vs. White: 0.73; 95% CI: 0.67–0.80), Hispanic (OR vs. non-Hispanic: 0.84; 95% CI: 0.77–0.93), or had at least mild symptoms of depression (OR vs. without: 0.90; 95% CI: 0.84–0.96) or anxiety (OR vs. without: 0.84; 95% CI: 0.78–0.90).

**Conclusions:**

Overall, 94% of PBHS participants remained enrolled after four years. Age, race, ethnicity, income, education, and symptomatic depression/anxiety were significantly associated with longitudinal remote questionnaire completion. These findings on engagement over time may inform future longitudinal study design.

**Clinical Trial Registration:**

Clinicaltrials.gov, identifier (NCT03154346).

## Introduction

Longitudinal cohort studies in the general population are important to understand health evolution, natural history of disease, and individual symptoms over time. In these studies, repeated measures provide high-quality data that track and evaluate health conditions, enabling inferences between exposures and outcomes (1). Participant selection, enrollment, and especially retention, are key to bias minimization, increased representativeness, and generalizability of results. These goals may be served by participant-centered study design features, but little data exists regarding factors that impact and influence long term retention and participation.

Use of remote digital support in longitudinal cohort studies holds much promise for research because of potential for participant-centered, scalable, and secure data collection. Such tools may facilitate efficient data collection and foster representativeness (2, 3). However, it is well-recognized that compliance is a major obstacle in observational studies, and this may be especially true for those that are conducted remotely (4). Little is reported about determinants of long-term remote engagement and participation (i.e., greater than 12–24 months) in remote, longitudinal observational studies, outside of retention and non-withdrawal (4). Increasingly, though, consideration of participant characteristics has been highlighted for optimal execution of such studies (5). Particular features of remote research are unique and may not be well-represented by cross-walking experience germane to in-person research participation. In particular, remote data collection, including through ePROs and digital health technologies, may be impacted by professional (e.g., employment status) or sociodemographic (e.g., access to technology) circumstances (3, 5). Much remains to be learned from multi-year remote studies, in which participants were not selected or motivated to participate based on particular disease state, condition, or risk factor. Overall, understanding factors associated with continued participation in remote research amongst a diverse set of participants can reinforce data quality, and is critical to promote equity in digitally-enabled research.

The Project Baseline Health Study (PBHS) is a longitudinal community-based multicenter observational cohort study of diverse participants designed to deeply characterize health and health transitions over time (6). The PBHS study was designed to include a range of participants across the health spectrum, from generally good health to varying levels of disease risk. The enrollment population for PBHS was stratified by age and sex to achieve a representative population with regard to race and ethnicity similar to the U.S. Census data. Because electronic surveys were sent on a quarterly basis, PBHS offers a unique chance to investigate aspects of remote study participation over an extended period of time in the context of a prospective longitudinal study.

In this analysis, we report 4-year retention and describe patterns of remote survey participation and completion rates among PBHS participants. We also investigate how demographic characteristics, social determinants of health, and health status are associated with remote survey completion.

## Methods

### Study participants

PBHS enrolled 2,502 adult residents of the United States between March 30, 2017 to April 26, 2019, and followed participants for up to 5 years; the final on-site follow-up visit occurred in 2023. Data were collected from a hybrid of annual clinical visits and annual, biannual, and quarterly remote assessments administered electronically. Further descriptions of study procedures and methods in PBHS have been published previously (6). The study design for the PBHS is provided in the Supplementary Figure S1.

**Figure 1:**
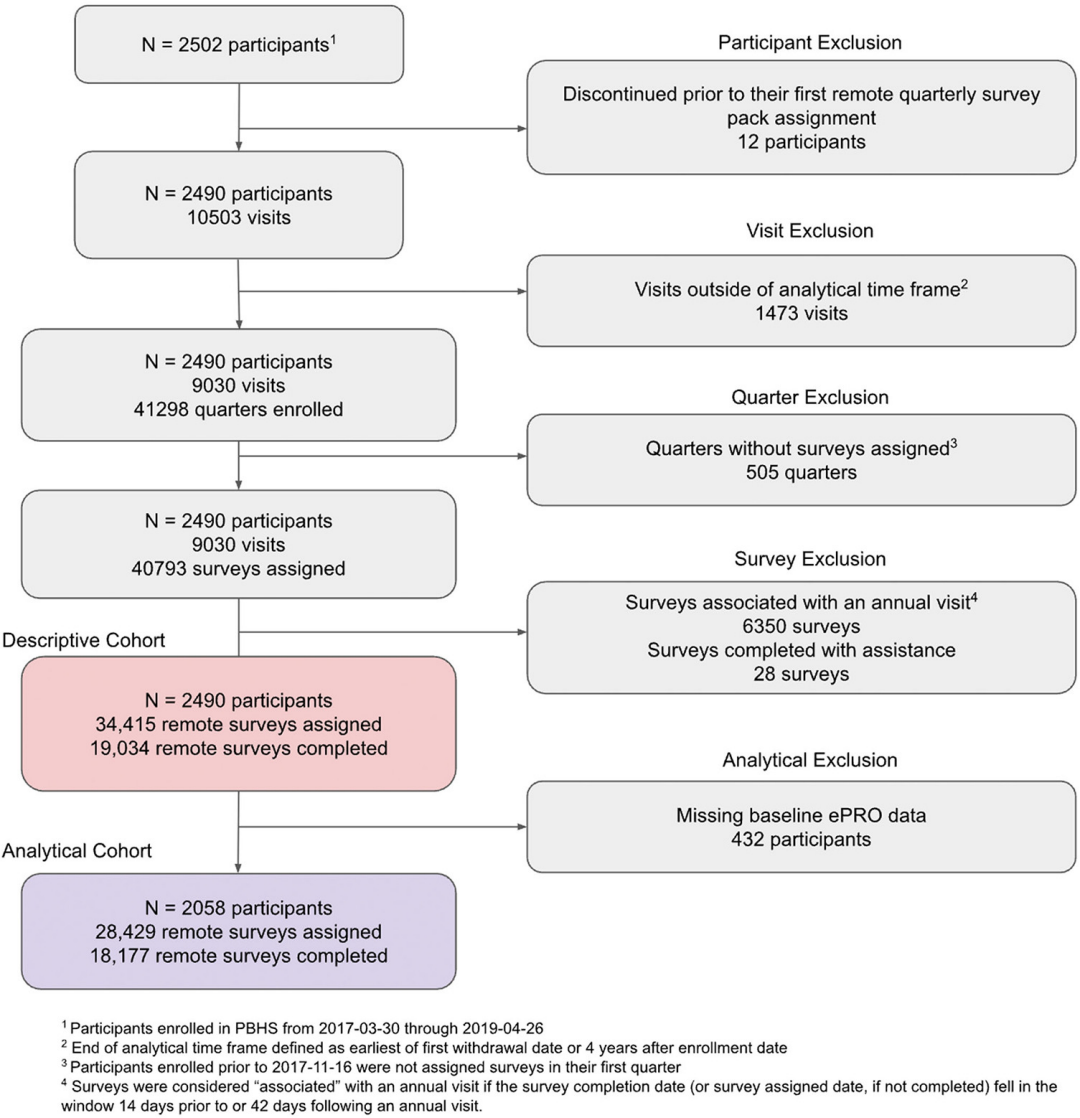
Participants and quarterly surveys included in analysis.

While all PBHS participants were eligible for inclusion, participants who withdrew from the study prior to initiation of remote data collection were excluded from this analysis. Subsequently, any study activities that occurred beyond the window extending from enrollment date to study exit or four years following enrollment, whichever was first, were also excluded. Figure 1 fully details eligibility criteria for this analysis.

Informed consent was obtained from all participants enrolled in PBHS and the study was approved by a central institutional review board (IRB; Western IRB) and the IRB at each of the participating institutions (Stanford University, Duke University, and the California Health and Longevity Institute). The PBHS was registered in clinicaltrials.gov (identifier NCT03154346).

### Remote app-based questionnaires: electronic patient reported outcomes (ePROs)

PBHS initiated electronic survey data collection on December 1, 2017. Each quarter following enrollment, participants were sent a set of questionnaires to be completed remotely via web portal and mobile app (7). The content of each set varied, as individual questionnaires were distributed on either a quarterly, semi-annual, or annual basis. Because these questionnaires were delivered and completed electronically and involved standardized assessment of patient-reported outcomes, they are also referred to as electronic patient reported outcomes (ePROs). Surveys included Positive and Negative Affect Schedule (PANAS) (7), Satisfaction with Life Scale (SWL) (8), Subjective Happiness Scale (SH) (9), Alcohol Use Disorders Identification Test-Consumption (AUDIT-C) (10), Patient Health Questionnaire-9 (PHQ-9) (11), Patient Reported Outcomes Measurement Information System (PROMIS) Pain Intensity Scale and Pain Interference Short Forms (12), Multidimensional Scale of Perceived Social Support (PSS) (13), Generalized Anxiety Disorder-7 (GAD-7) (14), World Health Organization Disability Assessment Schedule 2.0 (WHODAS 2.0) (15), and the mobile version of EuroQol-5 Dimension 5 Level (EQ-5D-5L) Quality of Life instrument (16). Additionally, participants made quarterly updates to their life circumstances (employment, marital status, etc.) via a “Life Circumstances and Habits” (CLIFE) module. A full schedule of ePRO administration is reported in the Supplementary Table S1. The time to complete the ePRO set varied based on the number of questionnaires assigned in that quarter, but targeted a median of 8 minutes. For the purposes of this analysis, an ePRO set was marked as “complete” when all quarterly and biannual questionnaires in the pack were submitted; annual questionnaires were excluded from this definition as they were initially paper-based.

Participants were compensated $10 per completed ePRO set, via an electronic wallet system. Participants were sent reminders to complete surveys via both push notification and email. ePRO sets were available in the app for a limited amount of time; the window was initially 14 days for completion, but was extended to 42 days starting in April 2021, following the COVID-19 pandemic. Survey packs that remained incomplete at the end of the window were closed and unable to be reopened at a later date.

### Remote quarterly completion of ePROs

In order to use PBHS data in a way that best evaluates the experience of remote digital survey completion, the outcome for this analysis is calculated as the proportion of quarters where assigned ePROs were completed. Once per year, participants were evaluated by onsite visit at their study site; in order to mitigate the potential influence of in-person interactions on remote questionnaire completion, only those survey packs administered in time periods that were not associated with an annual onsite visit (i.e., “remote ePRO sets”) were considered in the evaluation of questionnaire completion. ePRO sets were considered to be associated with an annual visit if they were assigned or completed by the participant inside the window from 14 days before through 42 days following the actual (not scheduled) study-site visit date. Additionally, to strictly assess self-directed ePRO completion, we excluded submissions that had help from a call center or site administration, at any time during the year. For each participant, we calculated the number of remote ePRO sets assigned between enrollment and the end of the analytic time frame, defined as the earliest date of loss to follow-up, withdrawal, death, or 4 years from their enrollment date. Participant engagement was defined as the proportion of remote ePRO sets fully completed from the total that were assigned, but not associated with an annual visit, during the four-year analysis window.

### Baseline characteristics of interest

Independent variables in this analysis were considered at the baseline visit; if participants did not report characteristics at baseline, values were imputed via next observation carried backward, for the first 13 months of enrollment. Characteristics included sociodemographic characteristics as well as measures of mental and physical health status. Mood symptoms were measured using GAD-7 and PHQ-9, validated questionnaires to measure symptoms of anxiety and depression, respectively. Both GAD-7 and PHQ-9 were dichotomized to “at least mild symptoms” vs. “no symptoms” in a generalized linear model (GLM). Objective physical health status was measured using the Charlson Comorbidity Index (CCI) (17), and participants were classified as “objectively healthy” if their CCI score was less than at least 80% of their PBHS peers, grouped by decade of age (<50, 50–59, 60–69, 70–79, ≥80). Participants' subjective or perceived health status was measured using EQ-5D-5L, a measure of health-related quality of life. A full listing of participant characteristics, their availability, and summaries of derivation or categorization is presented in Supplementary Table S2.

### Statistical analysis

Participant characteristics were described in the population with available baseline data, both overall and classified by level of completeness during remote quarters: no remote surveys completed, 1%–25%, 26%–50%, 51%–75%, and 76%–100% completion.

#### Handling of missing data

The modeling stage of this analysis required that participants complete at least one app-based survey that collected baseline characteristics of interest (CLIFE and PHQ-9) in their first 13 months of enrollment; those who did not complete either of these surveys were excluded from the analytical population, as described in Figure 1. Among those who were included in the analytic population, missing values of remaining covariates of interest, collected onsite, were estimated via multiple imputation using chained equations (MICE) (18) with 5 imputations for each of 5 iterations. A full description of data availability is described in Supplementary Table S2.

#### Generalized linear models

Age-adjusted univariable and multivariable GLMs using binomial distribution with a log-link (logistic regression models) were fit to estimate associations between baseline characteristics and long-term remote visit completion. Pooled regression estimates from the 5 imputed datasets were generated with their variance calculated using Rubin's rules (19). Analyses were done using R v4.2.2 (20). Multiple imputation and generalized linear modeling were performed using the mice package in R (21).

### Role of the funding source

Verily Life Sciences is the funding source for the PBHS and is responsible for data collection. Authors were fully responsible for the data analysis and interpretation presented herein and the writing of this article. The following individuals [MKC, SF, JB, EPS, SAS, SSS] had access to the raw data. Authors had access to the full dataset for the study, reviewed and approved the final manuscript for submission.

## Results

**Figure 2:**
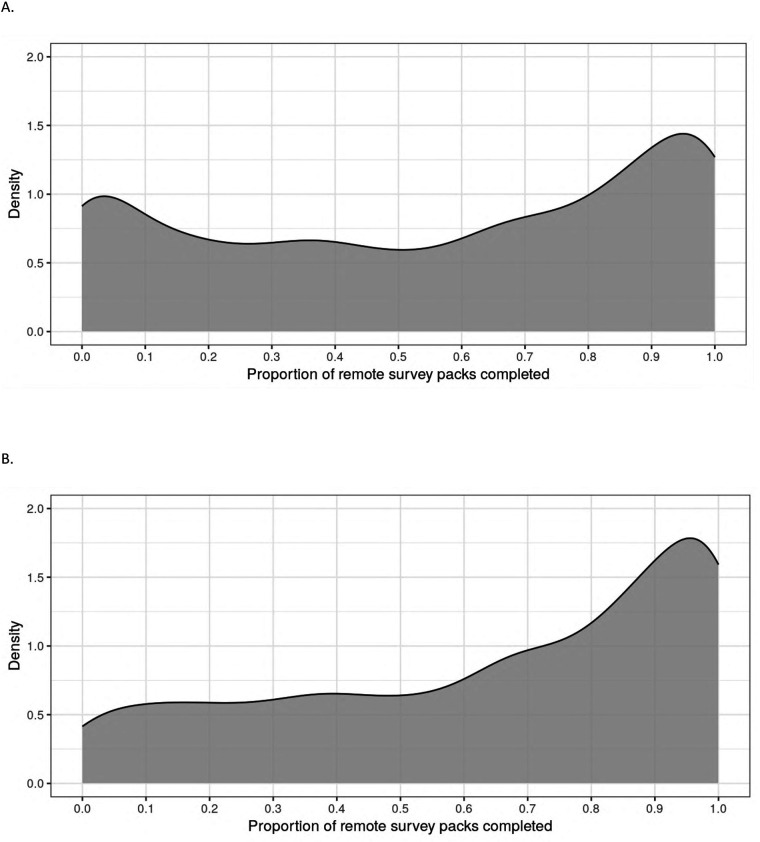
Density plot of proportion of remote survey packs completed within the first 4 years of study follow-up. **(A)** Participants who were ever assigned a remote survey pack (*N* = 2,490). **(B)** Participants who completed at least one remote survey pack within the first 13 months of enrollment (*N* = 2,058).

**Figure 3:**
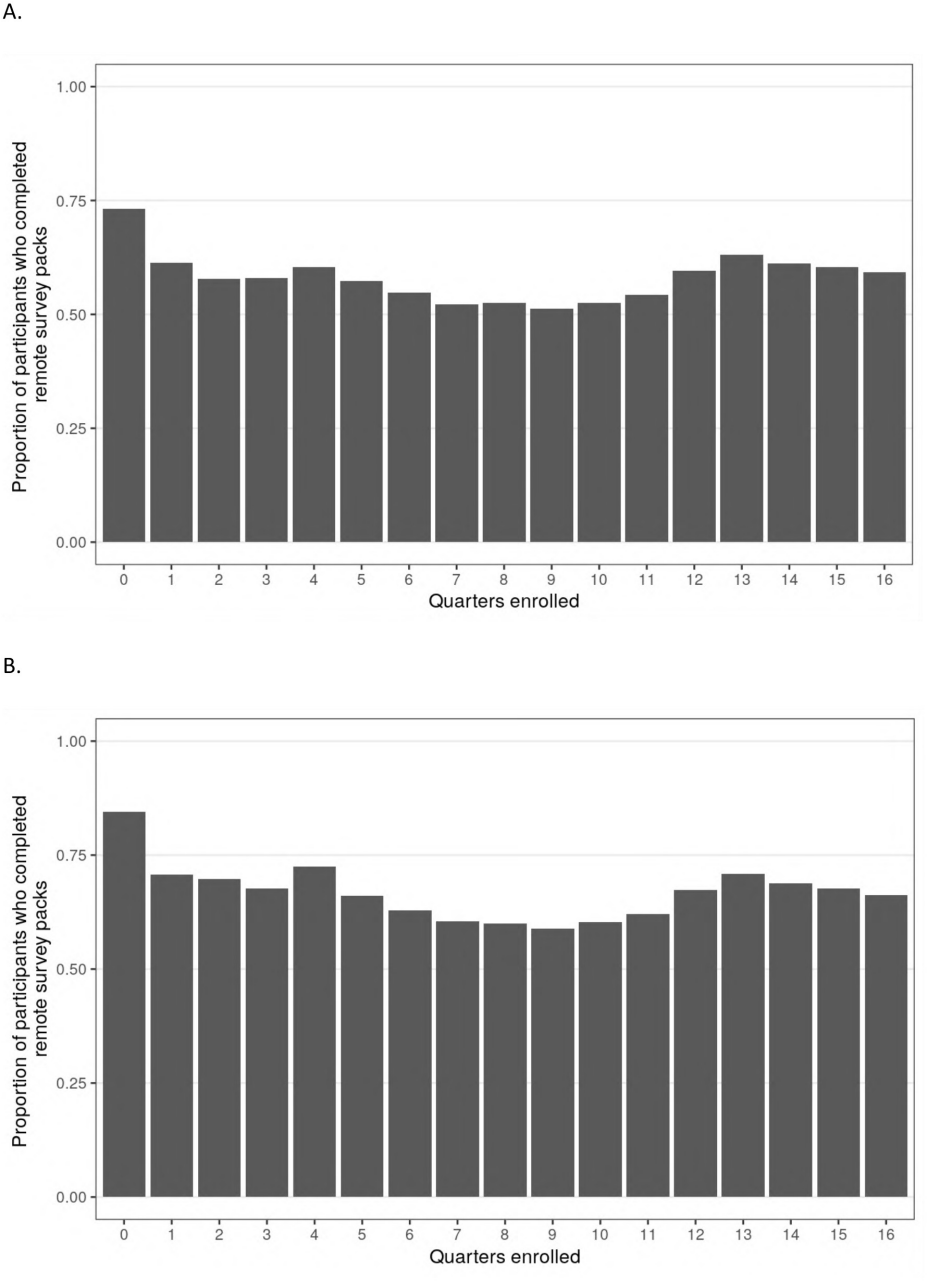
Chart A shows the proportion of participants completing remote survey packs over 16 quarters. Completion starts high at quarter 0, decreases, and rises again around quarters 13 to 15. Chart B follows a similar pattern but starts at a slightly lower completion rate in quarter 0, with a comparable decrease and subsequent rise around quarters 13 to 15.

Of the 2,502 participants who enrolled and completed a baseline visit in PBHS, 94% remained enrolled at the end of year 4. Additionally, 60% (*n* = 1,489) completed all 4 annual follow-up visits, while 80% (*n* = 2,010) completed at least 3 out of 4 annual visits, inclusive of visits during the COVID-19 pandemic when sites were closed. There were 2,490 participants who were enrolled in the study for at least one quarter; the median (IQR) number of remote ePROs assigned by the end of year 4 was 14 (13–15) of a maximum 16. The median (IQR) number of remote ePROs completed was 8 (3–12); 284 (11%) participants never completed a set of remote ePROs, while 373 (15%) participants completed 100% of the remote ePRO sets they were assigned. Figures 2A,B presents the distribution of remote ePRO completion within the first 4 years of study enrollment. Figures 3A,B describes the proportion of participants who completed remote survey packs by study quarter; at least 50% of participants completed remote ePRO packs in every quarter.

As detailed in Table 1, where distributions are reported in full, of the 2,058 participants in the analytic cohort, 82 (4%) participants never completed a remote survey set and 984 (48%) participants completed 76%–100% of surveys. Median ePRO-pack completion rate in the analytic cohort was 64% (IQR: 38%–93%).

**Table 1:** Baseline characteristics by proportion of survey pack completed (*N* = 2,058), including the demographics of the originating full-study cohort, for reference.

Characteristics		Full PBHS Cohort *N* = 2,502 (4)	Analytic Cohort (*N* = 2,058)	Proportion of remote survey packs completed				
				Never (*n* = 82)	>0–25% (*n* = 246)	26–50% (*n* = 308)	51–75% (*n* = 438)	76–100% (*n* = 984)
Demographics								
Median age, years (IQR)		49.9 (35.0, 64.0)	51.2 (36.0, 65.1)	37.7 (30.5, 50.4)	36.9 (28.1, 52.4)	45.0 (33.1, 59.8)	51.3 (36.4, 64.1)	57.3 (43.3, 69.1)
Female, *n* (%)		1,375 (55.0)	1,177 (57.2)	38 (46.3)	135 (54.9)	175 (56.8)	254 (58.0)	575 (58.4)
Race, *n* (%)	White	1,582 (63.2)	1,357 (65.9)	38 (46.3)	132 (53.7)	189 (61.6)	297 (67.8)	701 (71.2)
	Black or African American	400 (16.0)	289 (14.0)	20 (24.4)	41 (16.7)	59 (19.2)	57 (13.0)	112 (11.4)
	Asian	260 (10.4)	205 (10.0)	11 (13.4)	31 (12.6)	25 (8.1)	45 (10.3)	93 (9.5)
	Native Hawaiian or Pacific Islander	27 (1.1)	25 (1.2)	1 (1.2)	7 (2.8)	6 (2.0)	2 (0.5)	9 (0.9)
	American Indian or Alaska Native	31 (1.2)	24 (1.2)	2 (2.4)	3 (1.2)	3 (1.0)	2 (0.5)	14 (1.4)
	Other	201 (8.0)	158 (7.7)	10 (12.2)	32 (13.0)	26 (8.4)	35 (8.0)	55 (5.6)
Hispanic ethnicity, *n* (%)		290 (11.6)	243 (11.8)	17 (20.7)	45 (18.3)	49 (15.9)	46 (10.5)	86 (8.7)
Site, *n* (%)	Los Angeles	486 (19.4)	369 (17.9)	17 (20.7)	66 (26.8)	59 (19.2)	91 (20.8)	136 (13.8)
	Durham	487 (19.5)	371 (18.0)	17 (20.7)	40 (16.3)	51 (16.6)	62 (14.2)	201 (20.4)
	Kannapolis	518 (20.7)	448 (21.8)	15 (18.3)	40 (16.3)	66 (21.4)	89 (20.3)	238 (24.2)
	Palo Alto	1,011 (40.4)	870 (42.3)	33 (40.2)	100 (40.7)	132 (42.9)	196 (44.7)	409 (41.6)
Socioeconomic status								
Highest education, *n* (%)	High school or less	187 (9.0)	185 (9.0)	12 (14.6)	30 (12.2)	26 (8.4)	37 (8.4)	80 (8.1)
	Some college	505 (24.3)	500 (24.3)	19 (23.2)	63 (25.6)	82 (26.6)	109 (24.9)	227 (23.1)
	College	675 (32.5)	666 (32.4)	24 (29.3)	98 (39.8)	107 (34.7)	141 (32.2)	296 (30.1)
	Graduate degree or higher	704 (33.9)	701 (34.1)	27 (32.9)	53 (21.5)	92 (29.9)	149 (34.0)	380 (38.6)
	Prefer not to answer	6 (0.3)	6 (0.3)	0	2 (0.8)	1 (0.3)	2 (0.5)	1 (0.1)
Household income, *n* (%)	< $25,000	207 (10.0)	205 (10.0)	12 (14.6)	22 (8.9)	29 (9.4)	46 (10.5)	96 (9.8)
	$25,000–50,000	276 (13.3)	273 (13.3)	10 (12.2)	42 (17.1)	47 (15.3)	51 (11.6)	123 (12.5)
	$50,000–100,000	520 (25.0)	516 (25.1)	14 (17.1)	61 (24.8)	73 (23.7)	103 (23.5)	265 (26.9)
	$100,000–150,000	326 (15.7)	324 (15.7)	14 (17.1)	37 (15.0)	45 (14.6)	73 (16.7)	155 (15.8)
	$150,000–200,000	209 (10.1)	207 (10.1)	6 (7.3)	21 (8.5)	39 (12.7)	37 (8.4)	104 (10.6)
	> $200,000	385 (18.5)	380 (18.5)	21 (25.6)	44 (17.9)	56 (18.2)	97 (22.1)	162 (16.5)
	Prefer not to answer	154 (7.4)	153 (7.4)	5 (6.1)	19 (7.7)	19 (6.2)	31 (7.1)	79 (8.0)
Employment status, *n* (%)	Employed for wages	1,183 (52.4)	1,078 (52.4)	51 (62.2)	164 (66.7)	180 (58.4)	239 (54.6)	444 (45.1)
	Self-employed	268 (11.9)	236 (11.5)	15 (18.3)	29 (11.8)	34 (11.0)	57 (13.0)	101 (10.3)
	Student	61 (2.7)	57 (2.8)	2 (2.4)	15 (6.1)	13 (4.2)	9 (2.1)	18 (1.8)
	Homemaker	72 (3.2)	67 (3.3)	3 (3.7)	3 (1.2)	13 (4.2)	12 (2.7)	36 (3.7)
	Retired	469 (20.8)	441 (21.4)	3 (3.7)	15 (6.1)	40 (13.0)	87 (19.9)	296 (30.1)
	Not working <1 year	59 (2.6)	55 (2.7)	2 (2.4)	4 (1.6)	10 (3.2)	9 (2.1)	30 (3.0)
	Not working >= 1 year	52 (2.4)	50 (2.4)	1 (1.2)	8 (3.3)	8 (2.6)	9 (2.1)	24 (2.4)
	Unable to work	70 (3.1)	60 (2.9)	4 (4.9)	8 (3.3)	6 (1.9)	12 (2.7)	30 (3.0)
	Prefer not to answer	20 (0.9)	14 (0.7)	1 (1.2)	0 (0.0)	4 (1.3)	4 (0.9)	5 (0.5)
Health status								
Any history of smoking, *n* (%)		881 (35.2)	728 (35.4)	35 (42.7)	78 (31.7)	120 (39.0)	164 (37.4)	331 (33.6)
PHQ-9 score >4, *n* (%)		703 (30.1)	602 (29.3)	28 (34.1)	95 (38.6)	106 (34.4)	124 (28.3)	249 (25.3)
GAD-7 score >4, *n* (%)		622 (25.3)	500 (24.4)	26 (32.5)	86 (35.5)	87 (28.5)	105 (24.3)	195 (19.9)
Age-based CCI >80th percentile, *n* (%)		266 (13.2)	266 (13.2)	11 (13.9)	36 (15.1)	33 (11.1)	54 (12.4)	132 (13.6)
EQ-5D-5L Index <1, *n* (%)		1,374 (63.5)	1,170 (63.0)	37 (60.7)	140 (67.3)	157 (59.5)	257 (63.0)	579 (63.1)

IQR, interquartile range; PHQ, Patient Health Questionnaire; GAD, general anxiety disorder; CCI, Charlson comorbidity index; EQ-5D-5L, EuroQoL 5-domain 5-levels.

Table 1 describes sociodemographic and health status characteristics by proportion of ePRO packs completed. There was a positive univariate association between age and remote survey completion (median age: 38 years for >0%–25% completion vs. 57 years for 76%–100% completion). Participants who identified as White race or of non-Hispanic or Latino ethnicity were more likely to complete a greater proportion of ePROs (>0%–25% completion group was 54% White and 82% non-Hispanic or Latino vs. 71% White and 91% non-Hispanic or Latino in 76%–100% completion group).

Age-adjusted univariate and full multivariable logistic regressions were performed on the 2,058 participants whose baseline characteristics collected remotely were available, and the odds ratios (ORs) are presented in Table 2. Age was positively associated with remote survey pack completion after adjusting for all other covariates; participants who were older than 70 years of age had 2.56 (95% CI: 2.24–2.94) times greater odds of survey completion vs. participants who were 30 years of age or younger. Black participants had lower odds of completion (OR: 0.73; 95% CI: 0.67–0.80) vs. White participants, and Hispanic participants had lower odds of completion (OR: 0.84; 95% CI: 0.77–0.93) vs. non-Hispanic participants. Participants who were ever smokers had lower odds of completion (OR: 0.77; 95% CI: 0.72–0.82) vs. never smokers. Education was positively associated with remote survey completion (advanced degree vs. high school or less OR: 1.36; 95% CI: 1.22–1.52), while income was negatively associated with completion. Retired participants had significantly greater odds of completion vs. employed participants (OR: 1.50; 95% CI: 1.36–1.67). Participants with at least mild symptoms of depression (OR: 0.90; 95% CI: 0.84–0.96) or anxiety (OR: 0.84; 95% CI: 0.78–0.90) were at decreased odds for remote survey completion than those with no symptoms. There was no association between objective (based on CCI) or subjective (based on EQ-5D-5L) health status and survey completion.

**Table 2:** Odds ratios of survey pack completion from bivariable (age-adjusted) and multivariable generalized linear regression models (*N* = 2,058).

Variable		Bivariable models		Multivariable model	
		Estimate (OR)	95% CI	Estimate (OR)	95% CI
Age, years	≤30	—	—	1	1
	>30–40	—	—	1.516	(1.374, 1.672)
	>40–50	—	—	1.882	(1.700, 2.084)
	>50–60	—	—	2.218	(2.002, 2.458)
	>60–70	—	—	2.701	(2.413, 3.026)
	>70	—	—	2.563	(2.236, 2.938)
Female		1.141	(1.086, 1.200)	1.127	(1.065, 1.193)
Race	White	1	1	1	1
	Black or African American	0.793	(0.738, 0.852)	0.732	(0.674, 0.796)
	Asian	1.002	(0.921, 1.091)	0.931	(0.845, 1.026)
	Other	0.830	(0.764, 0.903)	0.911	(0.825, 1.008)
Hispanic ethnicity		0.840	(0.779, 0.906)	0.844	(0.768, 0.928)
Education	High school or less	1	1	1	1
	Some college	1.106	(1.006, 1.216)	1.028	(0.925, 1.141)
	College degree	1.136	(1.036, 1.246)	1.102	(0.988, 1.229)
	Advanced degree	1.341	(1.222, 1.471)	1.361	(1.215, 1.524)
	Prefer not to answer	0.729	(0.465, 1.138)	0.959	(0.555, 1.656)
Income	<$25,000	1	1	1	1
	$25,000–50,000	0.960	(0.866, 1.066)	0.768	(0.682, 0.864)
	$50,000–100,000	1.075	(0.978, 1.180)	0.797	(0.711, 0.892)
	$100,000–150,000	0.906	(0.818, 1.002)	0.600	(0.529, 0.679)
	$150,000–200,000	0.923	(0.825, 1.032)	0.628	(0.548, 0.719)
	>$200,000	0.755	(0.685, 0.833)	0.509	(0.449, 0.578)
	Prefer not to answer	0.897	(0.794, 1.014)	0.685	(0.594, 0.791)
Employment Status	Employed	1	1	1	1
	Not working	1.060	(0.966, 1.163)	1.165	(1.046, 1.299)
	Homemaker	1.193	(1.037, 1.376)	1.422	(1.209, 1.679)
	Student	1.059	(0.91, 1.232)	1.010	(0.849, 1.203)
	Retired	1.507	(1.368, 1.661)	1.502	(1.355, 1.665)
	Prefer not to answer	0.812	(0.611, 1.084)	0.770	(0.576, 1.036)
Any history of smoking		0.781	(0.741, 0.823)	0.766	(0.720, 0.815)
PHQ-9 sum score >4		0.791	(0.750, 0.835)	0.897	(0.836, 0.964)
GAD-7 sum score >4		0.782	(0.739, 0.829)	0.836	(0.776, 0.900)
Age-based CCI >=80th percentile		0.994	(0.923, 1.071)	0.963	(0.906, 1.023)
EQ-5D-5L index <1		0.911	(0.862, 0.962)	1.027	(0.947, 1.115)

CI, confidence interval; OR, odds ratio; PHQ, Patient Health Questionnaire; GAD, general anxiety disorder; CCI, Charlson comorbidity index; EQ-5D-5L, EuroQoL 5-domain 5-levels.

## Discussion

Overall, 94% of PBHS participants remained enrolled after four years and 88.5% of participants in the PBHS completed at least one set of remote (i.e., not associated with a study site visit), app-based study questionnaires that were delivered quarterly over the course of the four-year sampling frame for this study. In this diverse study population, increasing age had a significant effect on ePRO completion (i.e., older participants had higher odds of completion). Female sex and advanced education level were associated with greater odds of completion, while increasing income, Black race, Hispanic ethnicity, a history of smoking, and reported depression and anxiety symptoms were associated with lower odds of completion. Additionally, participants who did not work outside the home (retired, unemployed, or homemakers) were more likely to complete surveys. The PBHS study design was intentionally participant-centric, with features such as return of results, participant-only webinars with researchers on study progress, newsletters and updates, and multiple avenues for participation. In particular, PBHS allowed for participants to engage with remote questionnaires even if they never completed an annual follow-up visit. Despite the intervening COVID-19 pandemic, 60% of participants completed all of their annual visits, while 6.5% never returned for an annual follow-up visit, indicating a relatively high level of study engagement amongst this community-dwelling, generally healthy population.

There are few reports regarding completion rates of remote, electronically delivered PRO questionnaires in longitudinal prospective research studies. Recently, some large-scale longitudinal cohort studies have reported analyses of long-term remote survey adherence, though study populations and methodologies varied. For example, in the Millennium Cohort Study, a longitudinal cohort study of military personnel with both paper- and app-based follow-up surveys assessing health and well-being administered every three to five years (22), approximately 60% of eligible participants responded to the first follow-up survey, 70% responded to at least one follow-up survey, and 42% responded to every follow-up survey (23). Between 2006 and 2016, 82% of surveys were completed online. Researchers noted that the survey completion rate decreased at each consecutive follow-up wave of questionnaires. Though the study population has important differences from PBHS, there was overlap across the two studies in factors that were identified to be associated with survey completion.

While holistic survey completion rates for the National Institutes of Health's All of Us Study are not published as of this manuscript's final submission, available data suggests that, for the first 315,007 participants, response rates for surveys sent remotely 90 days after enrollment (on topics such as healthcare access, family history, personal medical history, and later COVID-19 experience) could be estimated at approximately 20%–32% (24, 25). More recent survey response rates estimated from publication and the study website suggest survey completion rates from 17% to 58% (26, 27). Consistent with PBHS findings, participants who were older, had higher income, or identified as White or non-Hispanic were more likely to contribute to optional surveys (25, 28, 29). All of Us also endeavors to enroll a diverse, representative cohort of study participants, but at a larger scale, and these estimated completion rates are somewhat lower than the remote survey completion rates of 50%–75% per quarter observed in PBHS. These differences could be related to the reinforcement of PBHS participant engagement through study procedures, including annual in-person visits, or to other study-specific design features; they may also be related to the overall size of All of Us, which may have led to a less personalized user experience at the participant level.

A critical point for longitudinal studies is the distinction between study retention and completion of study procedures. This difference is especially important when longitudinal studies feature a host of activities (i.e., in-person assessments, remote self-report assessments, wearables), wherein some activities may be completed and others not. A recent systematic review and meta-analysis reported study retention in longitudinal cohort studies to be 73.9% [standard deviation (SD) 20.1%] (1), though many of these studies involved populations with particular diagnoses. How active participants were in completing study procedures, which was the focus of this analysis, was not specified.

There is some indication that use of electronic platforms for health-related activities, whether clinical or in the context of research, may vary based on demographic characteristics. For example, the National Cancer Institute's HINTS study, which targets a representative U.S. sample, found that the main users of health apps were younger, had more education, were healthy, and had higher income (30). Importantly, though, only about 33% of enrolled participants responded (31), which underscores the key problems with bias, representativeness, and generalizability with such remote surveys.

Understanding whether (and how) the presence of medical and/or psychiatric comorbidities, or the overall quality of life status, impacts longitudinal completion of study surveys over time is also important. Depression, for example, is common in the U.S. general population, with 8.1% of adults having depression in a given 2-week period (32). It is not adequate to extrapolate from studies specifically launched in clinical populations of depressed individuals because presumably, these subjects have (1) been diagnosed by a professional; (2) overcome barriers to reach a clinical setting; and (3) are at least interested in research which may indicate openness to therapy. Those clinical populations are very different from individuals living in the community with underrecognized or untreated depressive symptomatology. Recent data from the REGARDS longitudinal cohort study indicates that baseline depressive symptoms were associated with later study withdrawal in that longitudinal cohort study with an overall median follow-up period of 11 years (33). On the other hand, in a clinical study of patients with bipolar disorder, adherence to electronic self-monitoring was higher among those with higher disease burden; in that study, overall adherence to weekly self-ratings was 78.5%, and women were more likely to belong to the third of participants who were labeled by study authors as having “perfect adherence” (34).

Advantages of the current study include the representative nature of the PBHS, which allows for in-depth analysis of demographic, quality of life, and mental and physical health factors associated with remote study procedure completion rates in a study population with relatively low withdrawal. There are also insights to glean regarding health equity and the use of digital tools at individual and community levels (35). Another strength of the study is the robustness and completeness of the dataset itself (Supplementary Table S2), which includes a rich variety of assessments and domains. This offered the opportunity to employ a separate machine learning approach using random forest methods, described in the Supplemental Material, where we found similar associations, bolstering the strength of our primary analyses and conclusions.

Caveats of the current analysis are related to the limited generalizability of our results to other, more decentralized, studies in terms of engagement or other critical study design characteristics. First, we have been able to document demographic characteristics (race/ethnicity, occupation, age) associated with engagement; however we acknowledge that the PBHS was not necessarily designed to explore the impact of socioeconomic issues such as health literacy, language barriers or technology access. Our study population was more affluent (based on household income) and more educated (based on proportion with college degree or higher) than the median in the US population, which may have biased any inferred associations of socio-economic status markers. Second, while we report on remote engagement aspects, the PBHS as a whole had a hybrid design. PBHS participants were scheduled to have in-person study visits and procedures annually, including personal contact with a study coordinator who may have answered questions about the remote application, or generally provided interpersonal contact. Of note, annual on-site study participation was consistent with remote study activity engagement; therefore it would be impossible in this case to disentangle any potential reinforcements of in person activities on remote engagement and vice versa. Thus, further investigation is warranted into the effect on remote engagement of the features unique to the hybrid design of PBHS, such as the effect of study coordinators and the study call center; these points are currently out of scope for this analysis. In addition to the in-person elements unique to a site-based or hybrid study, PBHS implemented outreach mechanisms such as emailed newsletters, app notifications, and other study-related events including educational seminars, which may help address engagement barriers in general. In concept, outreach resources can be managed in an adaptive fashion if and when engagement gaps emerge, but further research is required to quantify the impact of these outreach mechanisms on engagement in this population. It should be noted that these outreach features can be leveraged within the context of a fully decentralized study; doing so should be considered a critical part of a patient-centric approach to research with the potential added benefit of increasing engagement. Finally, this study was partly conducted during the COVID-19 pandemic, requiring annual visits to be delayed or adapted for off-site data collection. We did not study whether pandemic-related phenomena, including shifts in individual mobility or increased visibility into clinical research, had an effect on study retention or survey completion. All in all, we believe that the high levels of remote engagement can be largely attributed to the design of the remote components of the study, making the PBHS learnings valuable, but this study had several idiosyncrasies (intrinsic and pandemic-related) which call for additional research to pressure-test the generalizability of our findings. It should be noted that participants did receive remuneration for survey pack completion (approximately $10) which may have influenced completion rates to a degree that cannot be estimated using available data. Future research may consider remuneration when fiscally feasible, to the extent that this fair compensation for time and effort may have incentivized completion of participant tasks.

In conclusion, data from PBHS provide evidence that high rates of remote ePRO completion are achievable over four years among a population of diverse, community-dwelling, generally healthy adults. These results provide key evidence that prioritizing participant centricity can have measurable, important impacts on study retention and data quality. This study also found novel associations with remote PRO questionnaire completion, including that Black race, Hispanic ethnicity, smoking, symptoms of anxiety and depression, and increased income were associated with lower odds of completion, whereas increasing age and education level were associated with higher odds of completion. Understanding factors associated with remote questionnaire completion may inform future study design and participant engagement strategies, to help address barriers to participation among marginalized populations and to maximize study adherence and data completeness.

## Data Availability

The deidentified PBHS data corresponding to this study are available upon request for the purpose of examining its reproducibility. Interested investigators should direct requests to jsaiz@verily.com. Requests are subject to approval by PBHS governance.
