# Supplementary material for: Retention and characteristics associated with remote questionnaire completion in a general population cohort study: the project baseline health study
Source: Front Digit Health. 2025 Jun 24;7:1520132. doi: 10.3389/fdgth.2025.1520132 (PMC12235916; doi:10.3389/fdgth.2025.1520132)
Supplement: Supplementary file 1 [file Supplementaryfile1.docx]

# SUPPLEMENTAL MATERIAL

**Overall Retention and Characteristics Associated With Longitudinal Completion of Remote Patient-Reported Outcome Questionnaires in a Representative, General Population Cohort Study: The Project Baseline Health Study**

Megan K. Carroll MS; Safa Faheem BBA; Jean Bouteiller, MS; Adrian Hernandez MD; Kenneth W. Mahaffey MD; Jessica L. Mega MD; Neha Pagidipati MD, MPH; Terry Schaack MD; Svati H. Shah MD; Sumana Shashidhar MS; Susan Swope RN, CCRC, MS; Donna Williams RN, MSN, MPH; R. Scooter Plowman MD, MBA, MHSA; Edgar P. Simard PhD, MPH^;^ Sarah A. Short MPH; Shannon S. Sullivan MD, MSc

S Figure 1: Study Design, Project Baseline Health Study (from Arges et al, 2020).


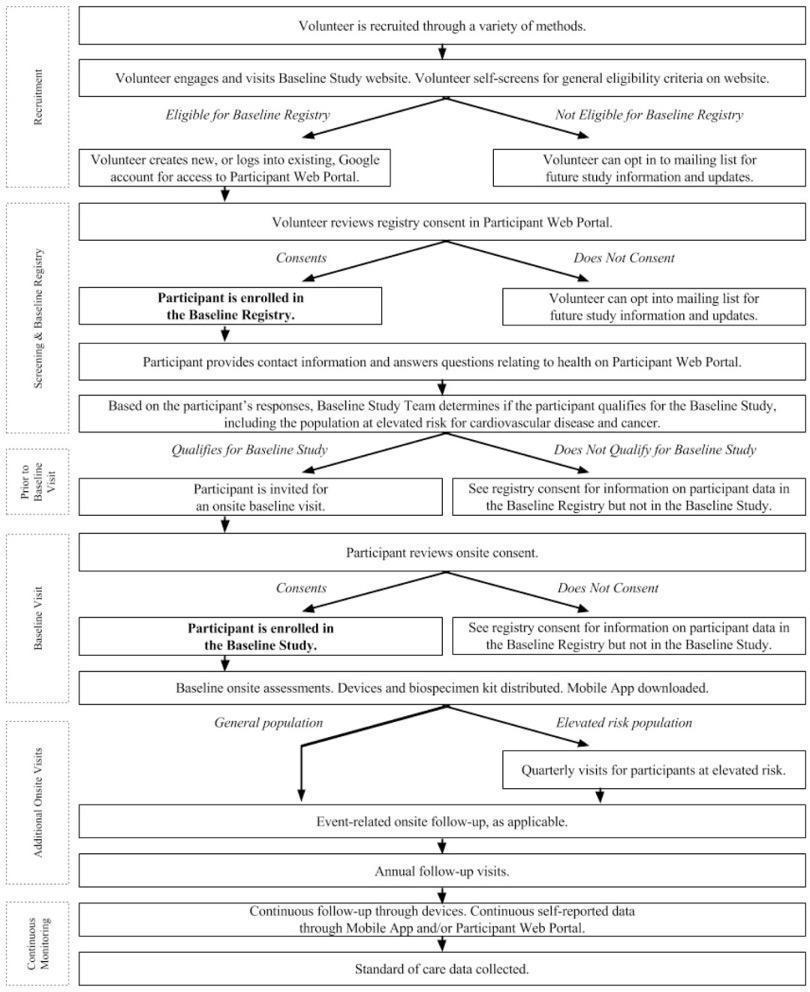


S Figure 2: Schematic* of Analyzed Quarters of Remote Electronic Patient Reported Outcome Questionnaires


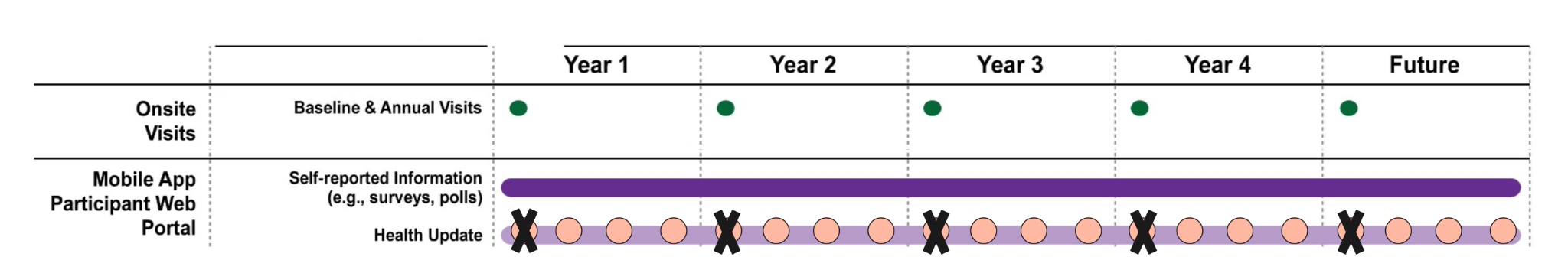


* This schematic describes the planned approach. If a participant missed an annual visit, then no remote surveys were excluded from analysis. Similarly, if the annual visit didn’t occur exactly one, two, etc. years after the baseline visit, then the survey associated with that actual annual visit timing would be excluded from analysis.

S Table 1: PBHS Mobile App-Based Questionnaires and Frequency

|  | **First Date of App-Based Collection** | **Last Date of Paper Collection** |
| --- | --- | --- |
| **Quarterly Surveys^1^** |  |  |
| PANAS | 2017-12-01 | NA |
| PSS | 2017-12-01 | NA |
| SH | 2017-12-01 | NA |
| SWL | 2017-12-01 | NA |
| **Semi-Annual Surveys^1^** |  |  |
| AUDIT-C | 2017-12-01 | NA |
| CLIFE | 2017-12-01 | NA |
| PHQ-9 | 2017-12-01 | 2018-09-12 |
| PROMIS Pain Intensity Scale and Pain Interference Short Forms | 2017-12-01 | NA |
| **Annual Surveys** |  |  |
| EQ-5D-5L | 2021-06-18 | NA |
| GAD-7 | 2021-05-03 | NA |
| WHODAS 2.0 | 2021-05-03 | NA |
| **Ongoing** |  |  |
| Health Update |  |  |
| AUDIT-C= Alcohol Use Disorders Identification Test-Consumption; PANAS=Positive and Negative Affect Schedule ; PSS=Perceived Stress Scale; SH=subjective happiness; SWL=satisfaction with life; CLIFE=Life Circumstances and Habits; PHQ-9=Patient Health Questionnaire-9; PROMIS=Patient Reported Outcomes Measurement Information System; EQ-5D-5L=EuroQol-5 Dimension 5-Level; GAD-7=Generalized Anxiety Disorder-7; WHODAS 2.0=World Health Organization Disability Assessment Schedule 2.0 | | |

^1^ These surveys were included in defining whether a set of ePROs was completed

S Table 2: Participant Reported Characteristics eligible for inclusion in models, and their availability, reported at baseline or within the first 13 months of study enrollment

| **Participant Reported Characteristic** | | **GLM Derivation** | **Random Forest**  **Derivation** | **Missing, n (%)** | |
| --- | --- | --- | --- | --- | --- |
|  |  |  |  | **Descriptive Cohort**  **(N = 2490)** | **Analytical Cohort**  **(n = 2058)** |
| **Demographics** | Age | Categorical:  <=30  >30-40  >40-50  >50-60  >60-70  ≥70 | Continuous | 0 | 0 |
|  | Sex | Binary: Female vs. Male | | 0 | 0 |
|  | Race | Categorical:  Asian  Black or African American  Other  White | Binary:  Asian vs. non-Asian  Black or African American vs. non-Black or African American  Other vs. non-Other | 0 | 0 |
|  | Ethnicity | Binary: Hispanic or Latino vs. non-Hispanic or Latino | | 0 | 0 |
| **Life Circumstances/ Habits** | Smoking Status | Binary:  Ever smoker vs. never smoker | Binary: Former Smoker vs is not a former smoker  Current smoker vs is not a current smoker | 0 | 0 |
|  | Education | Categorical:  High school or less  Some college  College  Graduate degree or higher | Binary:  High school or less vs. at least high school | 415 (17) | 0 |
|  | Income | Categorical:  <$25,000  $25,000-50,000  $50,000-100,000  $100,000-150,000  $150,000-200,000  > $200,000 | Binary:  <$25k/year vs. ≥$25k/year | 415 (17) | 0 |
|  | Employment Status | Categorical:  Employed  Not working  Homemaker  Student  Retired  Prefer not to answer | Binary:  Unemployed vs. not | 415 (17) | 0 |
| **Health Questionnaires** | GAD-7 (paper-based) | Binary: GAD-7 > 4 vs. ≤ 4 | Continuous | 29 (1) | 12 (1) |
|  | PHQ-9 (paper- or app-based) | Binary: PHQ-9 > 4 vs. ≤ 4 | Continuous | 152 (6) | 0 |
|  | EQ-5D-5L (paper-based) | Binary: EQ-5D-5L index = 1 vs. < 1 | | 327 (13) | 200 (10) |
| **Medical History** | Charlson Comorbidity Index | Binary:  Age-based CCI >= 80th vs. < 80th PBHS-specific percentile | | 61 (2) | 37 (2) |

S Figure 3: Random Forest^a^ approach to analysis: Significant Associations^b^ between participant characteristics and Longitudinal e-PRO Completion and Estimate of Effect Size (Shapley Value^c^)


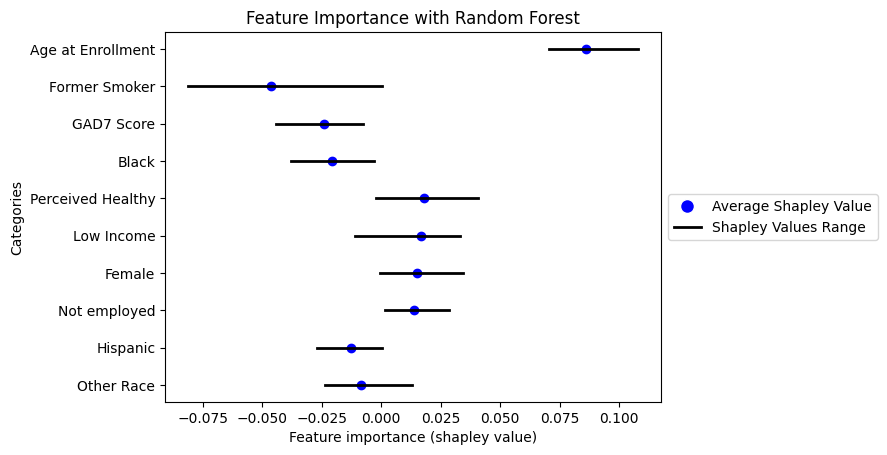


^a^For this random forest analysis, creation of n=100 random bootstraps with 50% of all participants was undertaken. For each bootstrap, we measured the Shapley value (SV) of each binary or continuous characteristic (‘feature’) with Random Forest Models. For each feature, we defined the Average Shapley value as the mean of all Shapley Values measured in these bootstraps. This value provides an estimate of treatment effect. If more than 95% of bootstraps reported a positive or /negative effect of the feature, we considered that the feature has a positive/negative effect on survey completion. If the 95% condition was not met, we considered that the characteristic did not have a statistically significant effect on survey completion. With this methodology, we also defined a minimum and maximum range of Shapley values for each feature.

^b^Note that participant characteristics not included in this plot were not statistically significantly associated with remote electronic questionnaire completion.

^c^ The Shapley value is a concept in cooperative game theory that assigns a fair and unique contribution to each feature in a model, reflecting their marginal impact on the outcomes when considering all possible permutations of features. Negative Shappley values indicate a negative effect on outcome. Positive Shapley values indicate a positive effect on outcome.
